# Effect of Different Disinfectants on Bacterial Aerosol Diversity in Poultry Houses

**DOI:** 10.3389/fmicb.2018.02113

**Published:** 2018-09-11

**Authors:** Linlin Jiang, Meng Li, Jinxiu Tang, Xiaoyu Zhao, Jianlong Zhang, Hongwei Zhu, Xin Yu, Youzhi Li, Tao Feng, Xingxiao Zhang

**Affiliations:** ^1^Ludong University School of Life Sciences, Yantai, China; ^2^Shandong Provincial Key Laboratory of Quality Safety Monitoring and Risk Assessment for Animal Products, Ji’nan, China

**Keywords:** high-throughput sequencing, disinfectant, microorganisms, 16S rRNA, poultry house

## Abstract

To better understand the effect of different disinfectants on the types and quantities of microorganisms in a broiler chicken house, five different types of disinfectants, including ozone, available chlorine, quaternary ammonium salt, glutaraldehyde, and mixed disinfectant, were used. The broiler house microbial communities were analyzed by high-throughput sequencing combined with air sampling. The results showed that the concentrations of airborne aerobic bacteria in the empty broiler houses after application of different disinfectants were significantly reduced compared to a house untreated with disinfectant (*P* < 0.05 or *P* < 0.01), and the number of inhalable particles of airborne aerobic bacteria sharply decreased after disinfection. Of the five disinfectants, the mixed disinfectant had the best disinfection efficacy on the total microbial communities (*P* < 0.05). A total of 508,143 high-quality sequences were obtained by high-throughput sequencing, which identified 1995 operational taxonomic units. In total, 42 phyla and 312 genera were identified. The structures of airborne microbial communities in the broiler houses after the different disinfectants were applied differed. In the house treated with the mixed disinfectant, the microbial communities containing opportunistic pathogens, such as *Escherichia-Shigella*, *Bacillus*, and *Pseudomonas*, had the lowest abundance, with a significant decrease compared to the house untreated with disinfectant. The alpha diversity index showed low diversity of the microbial communities in the house treated with mixed disinfectant. In contrast to the other four disinfectants, only a small amount of bacteria was detected in the air sample in the house treated with the mixed disinfectant; specifically, only four phyla were found (*Proteobacteria*, *Bacteroidetes*, *Actinobacteria*, and *Firmicutes*). The mixed disinfectant produced a positive effect on disinfection for four phyla; however, it didn’t thoroughly eliminate them. At genus level, *Bacillus*, *Arenimonas*, and *Shinella* could not be detected in the house treated with the mixed disinfectant, but were detected in houses treated with other disinfectants. The high-throughput sequencing results revealed that the combination of multiple disinfectants exhibited a good disinfection efficacy and that this technique could disinfect the air of broiler houses. These results will help guide the development of a reasonable program for broiler house disinfection.

## Introduction

In recent years, with the continuous improvement in the intensification of livestock husbandry and aquaculture, the density of housed animals has increased. In addition, the spread of animal infectious diseases is accelerating, and various animal epidemics have emerged (Wu et al., 2012; Mellata, 2013; Mughini-Gras et al., 2014; Threlfall et al., 2014; Barbosa et al., 2017; Poulsen et al., 2017; Stromberg et al., 2017; Vinayananda et al., 2017). Thus, the disinfection of the livestock and poultry houses has become an important measure to prevent and control diseases. The commonly used chemical disinfectants for broiler houses include available chlorine, ozone, quaternary ammonium salt, and glutaraldehyde (Meroz and Samberg, 1995; Saklou et al., 2016; Chidambaranathan and Balasubramanium, 2017). The different disinfectants used for large-scale disinfection of broiler houses operate via different mechanisms, and thus, their disinfection efficacies also differ (Suwa et al., 2013; Chidambaranathan and Balasubramanium, 2017; Maertens et al., 2017). Currently, chlorine-containing compounds (sodium dichloroisocyanurate, sodium hypochlorite, bleaching powder, chlorine dioxide, etc.) are widely used as disinfectants in livestock and poultry production (Van Klingeren, 1995; Boxall et al., 2003). Available chlorine can effectively kill *Mycobacterium tuberculosis*, *Enterobacter*, *Enterococcus*, *Staphylococcus aureus*, and *Bacillus subtilis var. niger* spores at a low concentration (Boxall et al., 2003; Hakuno et al., 2010; Suwa et al., 2013). Many studies have demonstrated that chlorine-containing compounds can kill microorganisms by altering their cell membrane structure (Venkobachar et al., 1977; Virto et al., 2005; Liu et al., 2006; Berg et al., 2010). Ozone is a triatomic gaseous molecule that acts as a powerful oxidant and has been used in medicine for more than 150 years (Elvis and Ekta, 2011). Ozone has broad medical applications due to its effective antimicrobial function (Ozturk et al., 2017) and antioxidant defense (Delgadoroche et al., 2017). Gram-negative bacteria are more sensitive to ozone than are Gram-positive bacteria, and bacteria are more sensitive than a yeast strain tested (Moore et al., 2000). Jacobs and Heidelberger (1915) synthesized for the first time a quaternary ammonium salt compound with bactericidal ability. The bactericidal mechanism of quaternary ammonium salts involves altering cell permeability, resulting in extravasation of the bacterial content. Quaternary ammonium salt is a cationic surfactant. Given that cations exhibit bactericidal effects based on lipophilicity and that the cell wall of Gram-positive bacteria contains more lipids than that of Gram-negative bacteria, Gram-positive bacteria are more easily inactivated by quaternary ammonium. Glutaraldehyde can act directly on bacterial proteins and enzymes and, thus affect bacterial metabolism and cause bacterial death. Glutaraldehyde can also prevent the release of dihydrochloride from the outer layer of the bacterial spores to prevent sporulation. Therefore, glutaraldehyde is applied more frequently for disinfection within broiler houses (Battersby et al., 2017; Castro et al., 2017). Glutaraldehyde exhibits a broad bactericidal spectrum with a highly efficient killing capacity for bacteria and virus. Glutaraldehyde also exhibits a strong effect on the spores generated by *Clostridium*, which can cause necrotic enteritis, and thus is commonly used for the disinfection of bacterial spores during epidemics (Miner et al., 1993; Rutala et al., 1993; Brantner et al., 2014).

During intense farming, livestock, and poultry can excrete bacteria and viruses, including opportunistic pathogens, through feces and the respiratory tract and generate bioaerosols in the air, which may harm humans and the environment (Hojovec and Fiser, 1968; Bessarabov et al., 1972; Petkov and Tsutsumanski, 1975; Petkov and Baĭkov, 1984; Petkov et al., 1987; Just et al., 2011a,b). After chickens are transferred or eliminated, the empty broiler house is left contaminated to varying degrees. Disinfection of empty houses is an important step in controlling diseases in large-scale chicken farms. Disinfection can reduce or kill potential pathogenic microorganisms in the house and prevent the transmission of pathogenic microorganisms between batches. Different disinfection procedures have varying results, so choosing appropriate disinfectants as well as disinfection procedures and methods can effectively control the occurrence of infectious diseases in poultry houses. These methods mainly involve cleaning, soaking, fumigating, spraying, and UV irradiating. Aerosolized disinfectants have been applied for more than half a century. Aerosol can conserve materials and form a disinfecting aerosol-disinfectant vapor gas system (Wichelhaus et al., 2006; Carvalho et al., 2015; Saklou et al., 2016). With the application of ultrasonic atomization technology, liquids can be atomized into an aerosol state to obtain uniformly dispersed 2–4 μm droplets. The atomized droplets play the role of “seed particles” and form an aggregation nucleus in the aerosol that can effectively collide with the surrounding fine particles to improve the aggregation efficiency. After the disinfectant is atomized, a large amount of water vapor is emitted in the air to increase the indoor relative humidity, improve the penetrating ability of the disinfectant on the bacterial wall, enhance the disinfection effect, and reduce the disinfection time (Muñoz et al., 2017).

The ideal disinfectant should have a good ability to kill pathogenic microorganisms; be stable in nature so that it is not easily affected by physical and chemical factors, such as water quality and organic matter; and effectively control the environmental sanitation of a chicken farm. Therefore, testing for microorganisms left after the disinfection of empty houses is an important means to evaluate the disinfection effectiveness. Numerous methods can be used for microbial aerosol detection, such as microbial culture counting, direct microscopic detection, biosensor technology, and gene chip technology. These methods are traditional and cannot accurately detect microorganisms that are not easy to culture or are present at low levels in the air. High throughput DNA sequencing methods can be used to characterize microbial systems via 16S rRNA sequence analysis. (Korajkic et al., 2015; Jia et al., 2016). The advantage of this technology is its ability to detect microorganisms that are difficult to obtain by culturing methods; therefore, this technology has been widely used in many fields (O’Brien et al., 2014; Tian et al., 2017). High-throughput sequencing technology can be used to evaluate the effect of disinfection on the distribution of microorganisms and the structure of the microbial communities in empty houses. In this study, five commonly used disinfectants were used to disinfect poultry houses. The composition of the microbiota present post-treatment was analyzed by high throughput sequencing of 16s rRNA and compared with the microbiota in an untreated house. The effects of different disinfectants on the number and diversity of microorganisms in the broiler houses when empty were investigated. This study provides a reference for the proper application of disinfectants in disease prevention and the development of disinfection procedures.

## Materials and Methods

### Sample Collection

The experiments were performed in Yantai, Shandong Province, China, in the winter of 2016. There were no residents or other farms located within 10 km of our experimental farm. The broilers were raised on a net, and the broiler houses were 100 m in length, 14 m in width, and 3.2 m in height. The chicken capacity of each broiler house was approximately 20,000. The temperature inside the house was 28°C, and the humidity was 65–70%. The houses were fully equipped with fans and wet curtains, longitudinal mechanical ventilation, and automatic feeding and water lines. The broilers had free access to feed and water. The ultrasonic atomization disinfection system (Shunxiang Farming Equipment Co., Ltd., China) was used in this study, and the disinfectant spray was prepared to a certain concentration using deep well water. The amount of disinfectant spray was calculated based on 15 ml/m^3^ of the broiler house. Five broiler houses were selected for the experiment, which were treated with five types of disinfectants: ozone (100 g/h, 1 h), available chlorine (1:1500, spray), quaternary ammonium salt (1:2000, spray), glutaraldehyde (1:1000, spray), and mixed disinfectant (including aldehydes, quaternary ammonium salt, and alcohol, 1:1500, spray). Bacterial aerosols in the broiler houses were collected. Samples collected after cleaning the broiler houses and rinsing the chicken manure were used for the untreated house sample data, whereas samples collected 72 h after disinfection of the broiler houses were used as the treated sample data.

### Detection and Analysis of Airborne Culturable Bacteria

**FIGURE 1:**
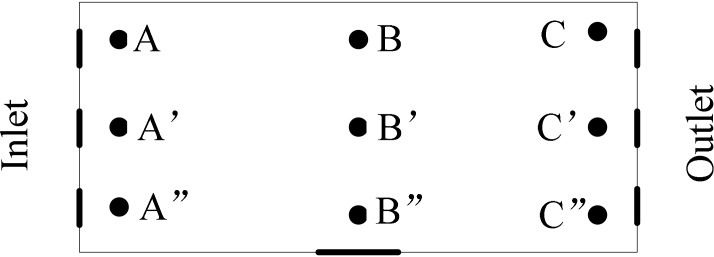
Layout of sampling sites of the tested broiler house.

Nine sampling sites were established in a broiler house with three sampling columns along the longitudinal direction of the broiler house. The two sampling columns on the left and the right were 1 m away from the two longitudinal walls of the broiler house and 6 m from the middle sampling column. Three sampling sites were evenly distributed in each sampling column and located 32 m away from each other (**Figure 1**). All the sampling heights were 0.5 m. Sampling was performed using an Andersen-6 air microbial collector (SKC, Philadelphia, PA, United States) (Andersen, 1958). In order to evaluate the detriment of bacterial aerosol to the approaching chicken in the nearest future in the treated house precisely, a Andersen-6 sampler was being utilized. The sampler simulated the anatomical and aerodynamic characteristics of the animal respiratory tract and was designed following the principle of inertial impact. Upon the principle of sampler, it can be evaluated that how big the bacteria particle that chicken inhaled is and in which part of the body the sedimentation took place. The sampler was divided into six stages with 400 holes per stage. The diameter of the holes was gradually reduced from the top to the bottom, as follows: stage 1, >7.1 μm; stage 2, 4.7 to 7.1 μm; stage 3, 3.3 to 4.7 μm; stage 4, 2.1 to 3.3 μm; stage 5, 1.1 to 2.1 μm; and stage 6, 0.65 to 1.1 μm. With an air flow rate of 28.3 L⋅min^−1^, the flow rate increased stage by stage so that the particles in the air were collected in the sampler Petri dishes at different stages according to the particle size. The airflow through the Andersen sampler was monitored using a mass flow meter (TopTrak 826; Sierra Instruments, Monterey, CA, United States). The air samples were collected from untreated broiler houses and those treated with five types of disinfectant under the following conditions: sampling time of 2 min, sampling medium of 5% defibrinated sheep’s blood soybean agar medium (purchased from Qingdao Hope Bio-technology Co., Ltd., China), 37°C and 24 h. The number of airborne colonies was calculated based on the following equation:

C=(N×1000)/(t×F),

where *C* represents the bacterial aerosol concentration in cfu⋅m^−3^; *N* is the number of colonies at each stage; *t* is the sampling time in min; and *F* is the sampling air flow in L⋅min^−1^.

### Sampling and Analysis of the Total Bacterial Aerosol in the Air

The total bacterial aerosol in the air was collected using a biosampler (SKC, United States). The sampling sites were 0.5 m above ground, and 50 mL of sampling solution (phosphate buffer saline, PBS) was placed in the sampling bottle. The airflow rate was 12.8 L⋅min^−1^, and the sampling time was 90 min. Ultracentrifugation was performed at 70,000 rpm⋅min^−1^ for 2 h. Total bacterial RNA was extracted using the EasyPure Viral DNA/RNA Kit (Beijing TransGen Biotech Co., Ltd., China). The reverse transcription kit EasyScript One-Step gRNA Removal and cDNA Synthesis SuperMix (Beijing TransGen Biotech Co., Ltd., China) was used for cDNA synthesis via reverse transcription, and the obtained samples were stored at −20°C. PCR amplifications were conducted with the primer set 515F/907R (515F: GTGCCAGCMGCCGCGGTAA, 907R: CCGTCAATTCCTTTGAGTTT), which targets the V4-V5 hypervariable region of the bacterial 16S rRNA gene (Chen et al., 2016, 2017; Guo et al., 2018). PCR reactions were performed in a volume of 30 μL containing 12 μL sterile water, 1.0 μL DNA template, 1.0 μL of each primer, and 15 μL 2 × Phusion^®^ High-Fidelity PCR Master Mix with GC Buffer (New England Biolabs, Ipswich, MA, United States). The PCR protocol was conducted with the following conditions: initial denaturation at 95°C for 3 min; 30 cycles of denaturation at 94°C for 30 s, primer annealing at 50°C for 1 min, and extension at 72°C for 1 min; and a final extension of 10 min at 72°C. Replicate amplicons were pooled for purification with a Qiagen Gel Extraction Kit (Qiagen, Germany). Finally, sequencing and construction of the 16S rRNA gene clone libraries were performed at Novogene (Beijing, China) using the Illumina HiSeq 2500 platform, and 250-bp paired-end reads were created. Three biological replicates were taken, respectively, at three sampling sites of each broiler house (both untreated and treated with a disinfectant).

### Sequence Processing and Analysis

Paired-end reads were trimmed from barcode and primer using self-written Python scripts and assembled by FLASH (Magoč and Salzberg, 2011). Quality filtering of data was performed using the Quantitative Insights Into Microbial Ecology (QIIME) software pipeline (Caporaso et al., 2010). The low-quality merged sequences (average quality value of <20) were removed from downstream analysis. Chimeric sequences were removed using UCHIME (Edgar et al., 2011). Reads that are singletons after quality filtering and global trimming are therefore discarded and reads with abundances of two or more are used as input for OTU clustering. Operational taxonomic units (OTUs) were determined with 97% similarity by UPARSE software (Edgar, 2013). For annotation of the 16S rRNA gene, the GreenGene Database ^1^ was used based on the RDP classifier^2^ algorithm to annotate taxonomic information. The relative abundance (%) of individual taxa within each community was estimated by comparing the number of sequences assigned to a specific taxon with the number of total sequences obtained for that sample. Alpha diversity was applied to analyze the complexity of species diversity for a sample through six indexes, including Observed-species, Chao1, Shannon, Simpson, ACE, Goodcoverage. All these indexes in our samples were calculated with QIIME 1.7.0 (Kuczynski et al., 2011). All raw sequences have been deposited in GenBank under accession number PRJNA464160.

### Statistical Analysis

Statistical analysis and plotting of the experimental data were performed with Excel (Microsoft, United States), SPSS 22.0 (IBM, United States), and GraphPad Prism 5 software (GraphPad Software, United States). The significance of the experimental data was processed using one-way analysis of variance (Majtán and Majtánová, 1999). A difference with *P* < 0.05 was considered significant, and a difference with *P* < 0.01 was considered extremely significant.

## Results and Analysis

### The Content and Particle Size Distribution of Aerobic Bacteria in Broiler Houses Treated and Untreated With Disinfectant

**FIGURE 2:**
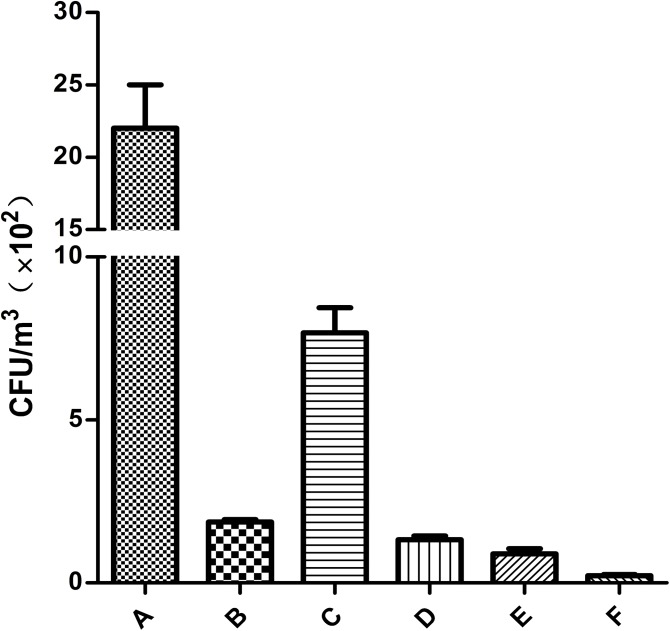
The total bacterial count in the air untreated and treated with disinfectants (*n* = 9). A: Without disinfection; B: ozone; C: available chlorine; D: quaternary ammonium salt; E: glutaraldehyde; F: mixed disinfectant.

The aerosol concentrations in the broiler houses that were untreated and treated with the disinfectants are presented in **Figure 2**. The treated houses had lower bacterial loads than the untreated house. After treatment with different disinfectants, the highest and lowest concentrations of bacterial aerosols were found in the broiler house treated with available chlorine and mixed disinfectant at 7.67 × 10^2^ cfu/m^3^ and 0.22 × 10^2^ cfu/m^3^, respectively. Obviously, the effectiveness of the mixed disinfectant on the cultured bacteria was superior to that of the other four disinfectants (*P* < 0.05).

As shown in **Table 1**, the distribution of the particle sizes of the airborne aerobic bacteria in the broiler houses treated with disinfectants changed significantly compared to those in the untreated house. The distribution of airborne aerobic bacteria in the broiler houses treated with different disinfectants decreased from stages III–VI of the Andersen-6 collectors. Compared with the empty house not treated with the disinfectants, the distributions in stages III–VI for the broiler houses disinfected with available chlorine, quaternary ammonium salt, glutaraldehyde, and mixed disinfectant were significantly reduced. In the broiler house treated with the mixed disinfectant, the proportion of airborne aerobic bacteria from stages V–VI significantly decreased from 35.1 to 0% compared with the proportion prior to disinfection, and glutaraldehyde decreased from 35.1 to 1.6%.

**Table 1:** The hierarchical distribution of bacteria untreated and treated with disinfectants.

Broiler houses	Stage	Airborne aerobic bacteria
Without disinfection	I, II 4.7–7.0 μm	28.3 ± 2.46
	III, IV 2.1–4.7 μm	36.6 ± 4.11
	V, VI 0.6–2.1 μm	35.1 ± 4.48
Ozone	I, II 4.7–7.0 μm	35.1 ± 3.68
	III, IV 2.1–4.7 μm	37.5 ± 2.50
	V, VI 0.6–2.1 μm	27.4 ± 1.56^A^
Available chlorine	I, II 4.7–7.0 μm	87.7 ± 1.83^a^
	III, IV 2.1–4.7 μm	4.6 ± 3.00
	V, VI 0.6–2.1 μm	7.7 ± 2.64^A^
Quaternary ammonium salt	I, II 4.7–7.0 μm	61.2 ± 4.78
	III, IV 2.1–4.7 μm	26.3 ± 3.21
	V, VI 0.6–2.1 μm	12.5 ± 2.32^A^
Glutaraldehyde	I, II 4.7–7.0 μm	72.3 ± 3.52^a^
	III, IV 2.1–4.7 μm	26.1 ± 2.00
	V, VI 0.6–2.1 μm	1.6 ± 1.53^A^
Mixed disinfectant	I, II 4.7–7.0 μm	1.0 ± 1.73
	III, IV 2.1–4.7 μm	99.0 ± 4.39
	V, VI 0.6–2.1 μm	0 ± 0^A^

*The difference in hierarchical distribution after the use of different disinfectants in the broiler house. “a” indicates P < 0.05; “A” indicates P < 0.01.*

### Sequence Analysis via High-Throughput Sequencing

After sequencing the V4–V5 (515F-907R) region of the bacterial 16S rRNA gene in airborne bacteria, the total number of raw sequences in the samples was 531,093. After filtering the low-quality sequences, the total number of effective sequences was 508,143. After the sequences were subject to quality control, the sequences were clustered into OTUs for taxonomic classification at 97% identity. All samples produced a total of 1995 OTUs (**Supplementary Table S1**), with an average length of 372 bp. The sequence information for each sample is presented in **Table 2**.

**Table 2:** Sequence information of the samples.

Sample name	Raw tags	Clean tags	OTUS
Without disinfection	84965	80990	668
Ozone	96289	92371	301
Available chlorine	83004	79775	636
Quaternary ammonium salt	90365	86715	614
Glutaraldehyde	90869	87388	240
Mixed disinfectant	85601	80904	95

### Microbial Community Structure Analysis

**FIGURE 3:**
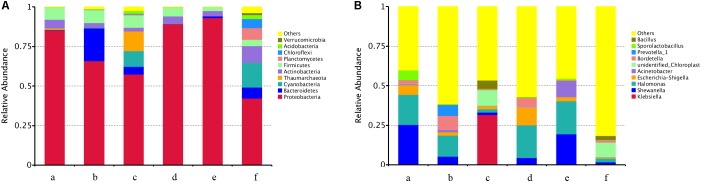
Community taxonomic composition and abundance distribution at the phylum level **(A)** and the genus level **(B)**. a, ozone; b, available chlorine; c, quaternary ammonium salt; d, glutaraldehyde; e, mixed disinfectant; f, without disinfection.

At the microbial taxonomy phylum level (**Figure 3A**), the airborne microorganisms collected in the broiler houses that were not treated or treated with five types of disinfectants mainly included Proteobacteria, Bacteroidetes, Actinobacteria, and Firmicutes. These phyla were dominant after treatment with ozone, available chlorine, glutaraldehyde, and mixed disinfectant, with relative abundances of 99.0, 97.9, 99.8, and 99.9%, respectively. The airborne microorganisms in the broiler houses that were not treated or treated with disinfectants differed at the phylum level. In total, 32 phyla were noted in the untreated house, whereas the numbers of phyla in the broiler houses treated with ozone, available chlorine, quaternary ammonium salt, glutaraldehyde, and mixed disinfectant were 21, 27, 28, 17, and 6, respectively. The relative abundance of OTUs was lowest after using the mixed disinfectant.

A total of 312 bacteria were detected at the genus level (**Figure 3B**). Among them, 218 bacterial genera were detected in the empty house without disinfectant, whereas the numbers of bacterial genera in the broiler houses treated with ozone, available chlorine, quaternary ammonium salt, glutaraldehyde, and mixed disinfectant were 140, 230, 213, 114, and 60, respectively. After treatment with different disinfectants, the distribution structures of the bacterial genera in the broiler houses differed. After application of the mixed disinfectant, the relative abundances of the opportunistic pathogens, such as *Escherichia-Shigella*, *Bacillus*, and *Pseudomonas*, were significantly reduced compared with those prior to disinfection.

### Microbial Community Diversity Analysis

The Chao1 index, ACE index, Shannon index, and Simpson index were used to assess the abundance and diversity of the microbial species in the samples (**Table 3**). The diversity indexes of different samples differed. The diversity index of the untreated broiler house was higher than the indexes of other houses, indicating that the biodiversity of the un-disinfected broiler houses was relatively rich. However, after treatment with the mixed disinfectant, the Chao 1 and ACE abundance indexes of the samples from the broiler house decreased compared with other samples. The lowest diversity index was noted for the mixed disinfectant, indicating that its disinfection effectiveness was superior to that of other disinfectants. Also, principal coordinate analysis (PCoA) showed a distinct clustering among the individual samples, and the sample from untreated house was clustered together and separated from the other treated houses samples (**Supplementary Figure S1**).

**Table 3:** Statistical analysis of bacterial alpha diversity in the samples.

Sample name	Shannon	Simpson	Chao1	ACE	Good coverage
Without disinfection	6.442 ± 0.96	0.965 ± 0.02	773.907 ± 137.13	792.510 ± 151.72	0.998 ± 0.00
Ozone	4.165 ± 0.77	0.879 ± 0.06	423.634 ± 203.55	451.296 ± 197.80	0.998 ± 0.00
Available chlorine	5.274 ± 0.16	0.933 ± 0.02	515.234 ± 206.71	538.657 ± 198.38	0.998 ± 0.00
Quaternary ammonium salt	5.074 ± 0.28	0.927 ± 0.02	594.562 ± 131.099	594.894 ± 161.46	0.998 ± 0.00
Glutaraldehyde	4.036 ± 0.54	0.855 ± 0.04	364.346 ± 79.463	421.425 ± 115.87	0.998 ± 0.00
Mixed disinfectant	3.587 ± 0.27	0.836 ± 0.03	186.436 ± 71.884	215.708 ± 94.712	0.998 ± 0.00

### Cluster Analysis

**FIGURE 4:**
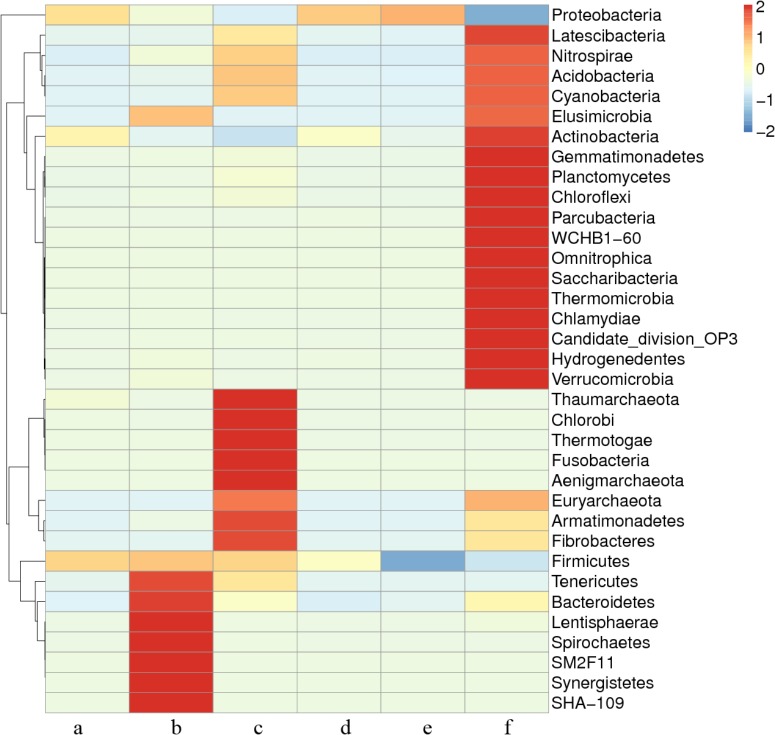
The relative abundance of bacteria at the phylum level. a, ozone; b, available chlorine; c, quaternary ammonium salt; d, glutaraldehyde; e, mixed disinfectant; f, without disinfection. The heatmap is color-coded based on row *Z*-scores. The scale bar was the standardized *Z*-value of the bacteria relative percentages in six samples. The color code indicates relative abundance, ranging from blue (low abundance) to yellow to red (high abundance).

A heat map (**Figure 4**) was constructed based on the 35 different taxonomies that can be classified at the phylum level. Each small box in the diagram represents the relative abundance of a certain phylum of bacteria in the sample. The more intense red color represents increased relative abundance. As shown in the heatmap, the composition of microbial communities in different samples differed. The abundance of the microbial communities in the air of the untreated broiler house was highest, and this abundance was obviously decreased after treatment with the combination of multiple disinfectants.

## Discussion

In this study, 16S rRNA high-throughput sequencing technology combined with the Andersen-6 microbial aerosol sampler (Andersen, 1958; Dalphin et al., 1991; Gołofit-Szymczak and Górny, 2010; Wu et al., 2017) was used for the in-depth investigation of the effect of different disinfectants on the concentration of bacterial aerosols; additionally, the effectiveness of the disinfectants and the risk of harm of microbial aerosols for people and livestock were indirectly evaluated. The number of colonies in the airborne bacterial culture was compared after disinfection. The results show that the concentration of bacterial aerosol was the lowest after disinfection with the mixed disinfectant. Therefore, the mixed disinfectant aerosol spray had the best efficacy. The other products, ranking in order of efficacy for bacteria, contained glutaraldehyde, quaternary ammonium salt, ozone, and available chlorine. Fate et al. (1985) used four commercial disinfectants in a poultry house to evaluate the efficacy for bacteria disinfection. The research of Fate et al. (1985) showed that the most effective disinfectant for reducing bacteria colony counts was a product that contained glutaraldehyde, compared with products containing cresylic acid, iodophors, and a combination of quaternary ammonium compound and formaldehyde. Our results also suggest that glutaraldehyde has good efficacy in disinfection of bacteria. The mixed disinfectant contained not only aldehydes but also quaternary ammonium salt and alcohol. This study found that the effect of the mixed disinfectant was superior to that of a single disinfectant. These results indicate that the mixed disinfectant has a significant effect on the airborne bacteria, demonstrating an obvious significance for the standardization of the use of disinfectants.

Currently, 16S rRNA high-throughput techniques are mostly used for analysis of microbial communities in the human gut and have rarely been applied for analysis of the structure of microbial communities of the microbial aerosol in livestock and poultry houses. Analysis of the structure of the microbial communities in the empty houses after treatment with five types of disinfectants revealed that disinfectants could effectively reduce the microbiological diversity and distribution of microorganisms in the broiler houses. At the genera and species levels, the number of detected OTUs was reduced. The effect of the multiple disinfectant combination was most obvious, followed by glutaraldehyde, and the effect of available chlorine was the worst. This finding is primarily attributed to the impact that the disinfection of chlorine disinfectant is easily affected by concentrations of available chlorine and environmental factors including pH, temperature, and organic matter (Russell, 1990; Frais et al., 2001; Rossifedele et al., 2011; Frazer et al., 2013). Thus, the disinfection effect was unstable. The mixed disinfectant was a combination of aldehyde, alcohol, and quaternary ammonium salt. The mixed disinfectant exhibited a broad antibacterial spectrum, strong bactericidal ability, and enhanced disinfection effect.

An effective disinfectant can disinfect the broiler house and its surrounding environment and protect against all viruses (enveloped and non-enveloped), bacteria, bacterial spores, and fungi. High-throughput technology is the most direct method to evaluate the bactericidal effect of disinfectants in studies of the bacterial community structure in the air of treated broiler houses. *Clostridium* and *Bacillus* are the main bacterial genera producing spores. Given that bacterial spores have a thick wall, most disinfectants are ineffective. Borick (Borick et al., 1964) and Starke (Stark et al., 1975) found that glutaraldehyde was capable of killing spores of *Bacillus* and *Clostridium* spp. within 3 h. The results of this study revealed that among the five types of disinfectants, glutaraldehyde, and mixed disinfectant could effectively clear *Clostridium*, and no bacteria of this genus were detected (**Supplementary Table S2**). Quaternary ammonium salt had the worst bactericidal effect on the spore-forming bacteria, with a detected concentration in air of 0.16%. *Bacillus* was not detected in the air of the broiler house treated with the mixed disinfectant. Glutaraldehyde exhibited the second lowest concentration, at 0.045%, whereas the broiler house disinfected with quaternary ammonium salt showed the highest concentration, at 5.11%. The reason why the mixed disinfectant was more effective than glutaraldehyde may be in the other compounds. Pepper and Chandler (1963) revealed that glutaraldehyde in an alcohol solution was superior as a sporicidal agent to both formaldehyde and glyoxal. As the mixed disinfectant contained aldehydes and alcohol, it may have a better sterilization effect. Our results revealed that the combination of multiple disinfectants had the best bactericidal effect on spore-forming bacteria, followed by glutaraldehyde, whereas quaternary ammonium salt had the poorest disinfection effect on spore-forming bacteria.

There are many opportunistic pathogens in bacterial aerosol. Choosing reasonable disinfectants can effectively block the spread of conditioned pathogenic bacteria. *Ochrobactrum*, *Escherichia-Shigella*, and *Bacillus* are three common opportunistic pathogens. After treating the chicken houses with different disinfectants, *Ochrobactrum* and *Bacillus* were not detected in the samples from the house treated with the mixed disinfectant (**Supplementary Table S2**). Second, the levels of these three bacteria after glutaraldehyde disinfection decreased significantly compared with those after treatment with the other disinfectants. *Ochrobactrum* and *Escherichia-Shigella* are both Gram-negative pathogenic bacteria, while *Bacillus* is a Gram-positive bacterium. These results showed that the mixed disinfectant had a better disinfection effect on conditioned pathogens, whether Gram-negative or Gram-positive bacteria.

The resistance of bacteria to disinfectants has also been a focus of research in recent years. The earliest discovered resistant bacterium is the conditional pathogen *Pseudomonas*, exhibiting resistance to a variety of disinfectants, including quaternary ammonium salt, chlorhexidine acetate, iodine, and phenol (Gilbert and Brown, 1978; Craven et al., 1981; Brozel and Cloete, 1993). The results of this study revealed that the combination of disinfectants had the best disinfection effect for *Pseudomonas*, followed by glutaraldehyde. The effect of quaternary ammonium salt was the worst. Previous studies demonstrated that bacterial resistance will gradually increase with the use of a disinfectant. However, after switching to other disinfectants, this resistance will disappear. This study also demonstrated that the combination of multiple disinfectants could overcome the resistance of bacteria to a single disinfectant during house disinfection. In the house treated with mixed disinfectant *Planctomycetes*, *Acidobacteria*, and *Gemmatimonadetes* were thoroughly eliminated, whereas the other disinfectants did not completely eliminate these bacteria. At genus level, the house treated with mixed disinfectant showed thorough elimination of *Bacillus*, *Arenimonas*, *Shinella*.

Although the ingredients of the mixed disinfectant were commonly used disinfectants, their concentrations and the methods used the disinfection process should be explored based on the conditions of the farm to achieve disinfection while reducing drug resistance. Besides, we were analyzing the DNA sample of bacteria in the air, whereas the sequence analysis does not differentiate between live and dead bacteria. As a result, while implementing sequencing, some certain OTUs might turn up in the house treated with disinfectant. These OTUs didn’t show up in the untreated house. These bacteria with low abundance can make a difference to the accuracy of analyzing data. Therefore, it is significantly important to search for new approach to evaluate the changes of microbial consortia structure accurately. The results revealed that the research and development of mixed disinfectants should be continuously strengthened to improve the disinfection spectrum, reduce the dosage, and enhance the safety for the staff and animals.

## Author Contributions

XXZ and LJ designed the experiments. LJ, JT, ML, and XYZ carried out the experiments. JZ, YL, and TF analyzed the experimental results. LJ, HZ, and XY wrote the manuscript.

## Conflict of Interest Statement

The authors declare that the research was conducted in the absence of any commercial or financial relationships that could be construed as a potential conflict of interest.

## Footnotes

1 http://greengenes.secondgenome.com

2 http://sourceforge.net/projects/rdp-classifier/
