# Supplementary material for: Effect of Different Disinfectants on Bacterial Aerosol Diversity in Poultry Houses
Source: Front Microbiol. 2018 Sep 11;9:2113. doi: 10.3389/fmicb.2018.02113 (PMC6142877; doi:10.3389/fmicb.2018.02113)
Supplement: Supplementary file 1 [file Table_1.DOCX]

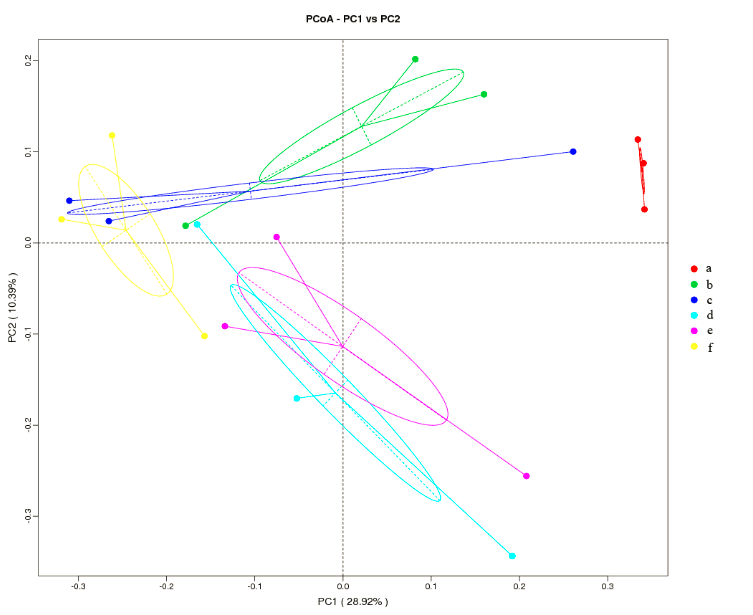


FIGURE S1 The principal coordinate analysis (PCoA) derived from the Bray-Curtis dissimilarity matrices based on the 97% OTU level of the bacterial community compositions at six samples. a, without disinfection, b, ozone; c, available chlorine; d, quaternary ammonium salt; e, glutaraldehyde; f, mixed disinfectant;.

Table S1. Sequence information of the samples

Table S2. Proportion of part of bacteria
